# ACE2 overexpressing mesenchymal stem cells alleviates COVID-19 lung injury by inhibiting pyroptosis

**DOI:** 10.1016/j.isci.2022.104046

**Published:** 2022-03-10

**Authors:** Jinhuan Wei, Rui Shang, Jiaqi Wang, Shengze Zhu, JianQiang Yin, Ying Chen, Yayu Zhao, Gang Chen

**Affiliations:** 1 Center for Basic Medical Research, Medical School of Nantong University, Nantong, Jiangsu Province 226001, China; 2 Department of Histology and Embryology, Medical School of Nantong University, Nantong, Jiangsu Province 226001, China; 3 Genesis Stem Cell Co. LTD, Nantong, Jiangsu Province 226009, China; 4 Key Laboratory of Neuroregeneration of Jiangsu and the Ministry of Education, Co-innovation Center of Neuroregeneration, Nantong University, Nantong, Jiangsu Province 226001, China; 5 Department of Anesthesiology, Affiliated Hospital of Nantong University, Nantong, Jiangsu Province 226001, China

**Keywords:** Biological sciences, Microbiology, Cell biology, Stem cells research

## Abstract

Mesenchymal stem cells (MSCs) have shown some efficacy in the COVID-19 treatment. We proposed that exogenous supplementation of ACE2 via MSCs (ACE2-MSCs) might have better therapeutic effects. We constructed SARS-CoV-2 spike glycoprotein stably transfected AT-II and Beas-2B cells and used SARS-CoV-2 spike pseudovirus to infect hACE2 transgenic mice. The results showed that spike glycoprotein transfection triggers the release of apoptotic bodies and formation of membrane pores in pyroptosis. Inflammatory factors and pyroptosis factors were highly upregulated by spike glycoprotein transfection. SARS-CoV-2 spike pseudovirus worsened lung injury and increased the main factors of cytokine storm and pyroptosis. Compared to using MSCs or rh-ACE2 alone, the administration of ACE2-MSCs could significantly reduce these factors better and alleviate lung injury *in vivo* and *in vitro*, which might be because of the increased activities of secretory ACE2. Our proposal is a promising therapeutic solution for preclinical or clinical research.

## Highlights

• The spike glycoprotein of SARS-CoV-2 induced pyroptosis in AT-II and Beas-2B cells
• SARS-CoV-2 spike pseudovirus-infected hACE2 transgenic mice caused lung injury
• HUC-MSCs alleviated lung injury by inhibiting pyroptosis and cytokine storms
• ACE2-MSCs has better therapeutic effects than using soluble ACE2 or MSCs alone

## Graphical abstract

**Figure:**
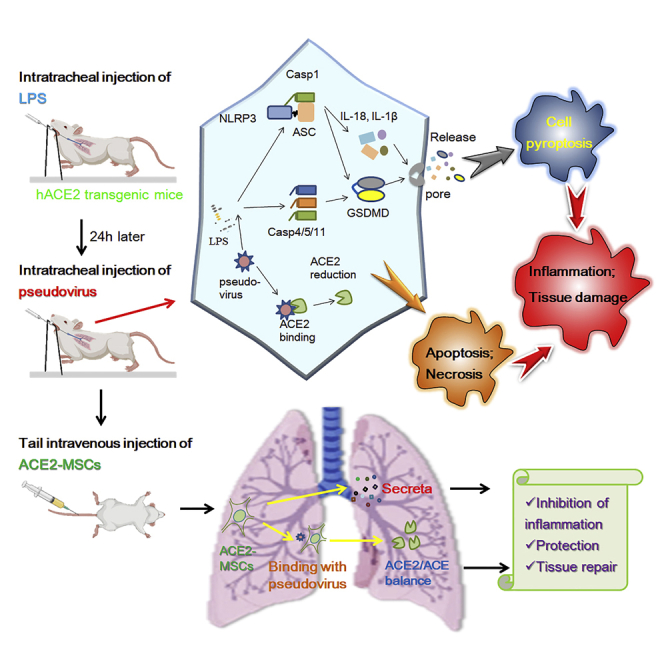

## Introduction

After the outbreak of SARS-CoV-2 (2019-nCoV), the number of new confirmed cases and deaths have increased worldwide. To date, according to records from the World Health Organization (WHO), more than 4,700,000 people have died from SARS-CoV-2. The virus triggers PANoptosis (pyroptosis, apoptosis, and necroptosis) (Frühbeck et al., 2021; Karki et al., 2021; Lee et al., 2020) and cytokine storms, which can cause tissue damage and mortality (Chen et al., 2020; Huang et al., 2020). Every country is working hard to fight SARS-CoV-2 and has developed COVID-19 vaccines approved by the WHO, which significantly reduced the infection rates and mitigated the symptoms. However, with the emergence of many mutations, no one can guarantee that the current COVID-19 vaccines are effective. Given the significant number of COVID-19 patients still present, it is urgent to cure these patients. Therefore, proper therapy is of utmost priority.

Based on the idea that MSCs not only have anti-inflammatory capabilities but may also be genetically engineered to carry target genes or medications, MSC-based therapy has been proposed as a valuable therapeutic technique for acute respiratory distress syndrome (ARDS) and acute lung injury (ALI) (He et al., 2015b; Khoury et al., 2020). Previous clinical trials have proven that intravenously injected MSCs highly accumulate in the lung and exert immunomodulatory effects to protect alveolar epithelial cells, prevent pulmonary fibrosis, remodel the pulmonary microenvironment, and adjust lung dysfunction (Qu et al., 2020; Wick et al., 2021). Therefore, a dozen scientists and medical experts considered MSC-based therapy a promising approach for treating COVID-19 patients. They designed and conducted clinical trials and finally obtained certain curative effects. Some groups performed phase 1 and phase 2 trials using human umbilical cord mesenchymal stem cells (hUC-MSCs) to treat severe COVID-19 patients and proved that hUC-MSCs treatment is safe and well-tolerated for moderate and severe patients (Meng et al., 2020; Shi et al., 2021). Some groups assessed that hUC-MSC infusions dramatically increased phase 1/2a COVID-19 patient survival and serious adverse event (SAE)-free survival and shortened the recovery time (Kouroupis et al., 2021; Lanzoni et al., 2021). Similarly, the clinical symptoms and inflammatory cytokines of severe COVID-19 patients with intravenous transplantation of hUC-MSCs return to the normal range faster than those without hUC-MSC treatment (Shu et al., 2020). The survival rate was increased in ill COVID-19 patients with comorbidities, and IL-6 was significantly decreased in recovered patients (Dilogo et al., 2021).

ACE2 is both a membrane surface receptor protein and a secretory protein that plays a protective role in treating lung injury. Human angiotensin-converting enzyme 2 (ACE2) is the attachment receptor for the spike glycoprotein (S protein) of SARS-CoV-2 that helps the virus enter humans, recombinant ACE2-Ig neutralized virus pseudotyped SARS-CoV-2 spike protein *in vitro* (Lei et al., 2020), and soluble human ACE2 was able to inhibit COVID-19 infections (Monteil et al., 2020). On the other hand, the dysfunctional balance of ACE/ACE2 is one of the main factors that lead to COVID-19-induced lung injury; therefore, compensation of ACE2 to balance ACE/ACE2 function should be a promising way to alleviate COVID-19-induced lung injury. Because hUC-MSCs negatively express ACE2 (Hernandez et al., 2021; Leng et al., 2020), overexpressing ACE2 in MSCs (ACE2-MSCs) could rescue lipopolysaccharide (LPS) or bleomycin-induced lung injury (He et al., 2015a; Zhang et al., 2015). Here, we aimed to discover whether overexpressing ACE2 in hUC-MSCs would be much more effective in treating COVID-19 patients than using MSCs alone.

Alveolar type II epithelial cells (AT-II) and bronchial epithelial cells (Beas-2B) are the main cell types in the lung and are considered to be the main target cells of COVID-19 because they highly express ACE2 and tyrosine-protein kinase receptor UFO (AXL), respectively. Strict biosafety regulations limit many studies because of the high risk of working with viruses. One alternative strategy is using recombinant virus or pseudovirus proteins for multiple immunizations to induce cellular and humoral immunity, which partially mimics the histopathological changes caused by viral infection (Chen et al., 2021; Liu et al., 2020). We mirrored the phenomena of COVID-19 in AT-II cells and Beas-2B cells by S protein transfection and in hACE2 transgenic mice by SARS-CoV-2 pseudovirus infection and then treated the cells and mice with MSCs alone or ACE2-MSCs. The results demonstrated that ACE2-MSCs have better therapeutic effects than MSCs alone to alleviate COVID-19-induced lung injury by inhibiting pyroptosis.

## Results

### Spike glycoprotein of SARS-CoV-2-transfected AT-II and Beas-2B cells induced pyroptosis and apoptosis

**Figure 1:**
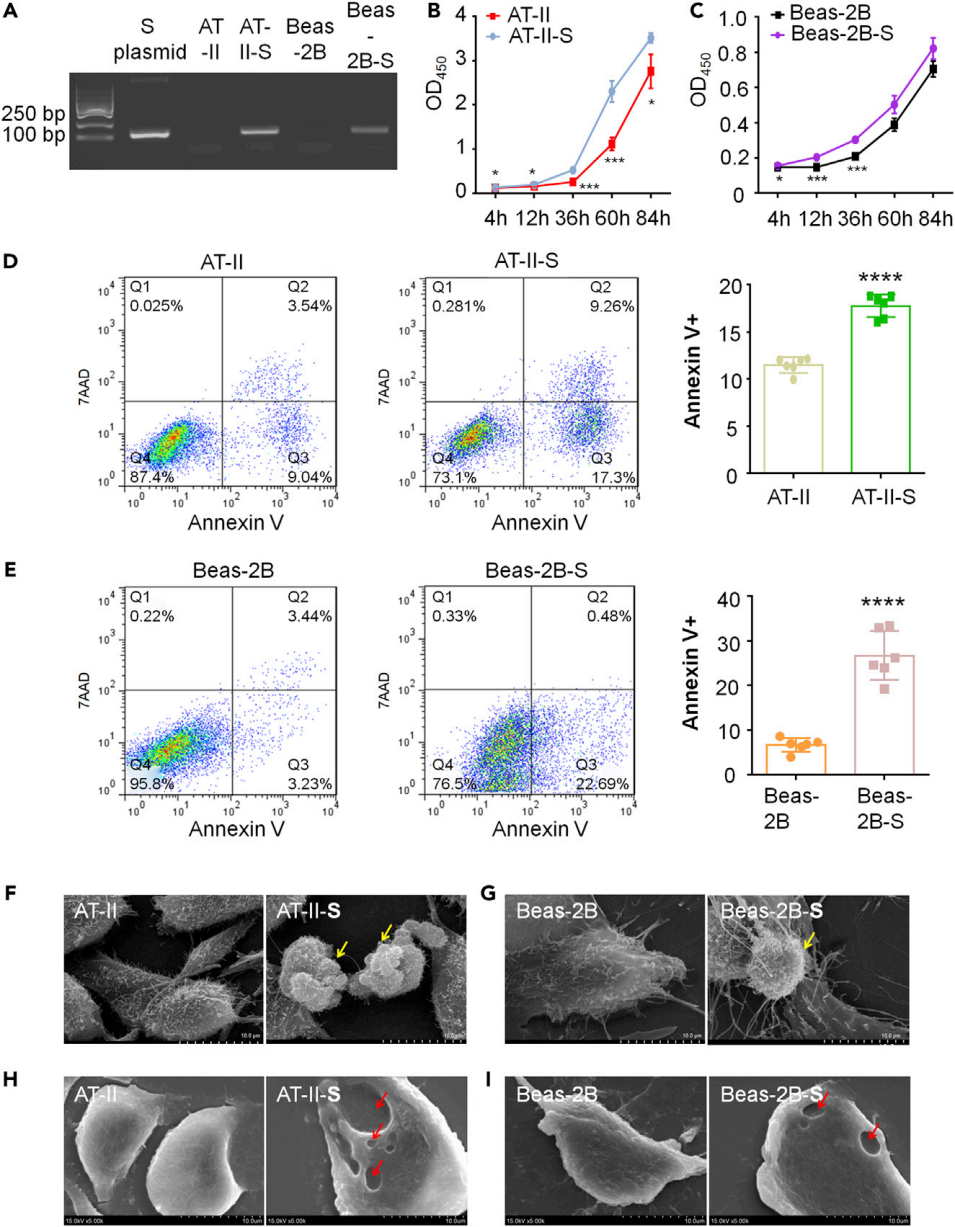
Spike glycoprotein of SARS-CoV-2-transfected AT-II and Beas-2B cells induced pyroptosis and apoptosis (A) PCR results of AT-II and Beas-2B cells stably transfected with SARS-CoV-2 spike glycoprotein, named AT-II-S and Beas-2B-S, respectively. The length of the PCR product is 129 bp. (B and C) Measurement of cell proliferation of AT-II-S and Beas-2B-S by CCK-8 4 h (4 h), 12 h (12 h), 36 h (36 h), 60 h (60 h), and 84 h (84 h) after cell planking compared with the untransfected groups. At each time point, we measured 4 replicate cell wells. All data are expressed as the mean ± SD. Student’s *t* test was used to compare the differences between groups, followed by Bonferroni’s test. ∗p < 0.05, ∗∗∗p < 0.001 versus control group. (D and E) Cell apoptosis was determined in AT-II-S and Beas-2B-S cells at 36 h after the cell planking time point via flow cytometry with Annexin V-7AAD and compared with the untransfected groups. Cells were collected from three replicate cell wells for each group. All data are expressed as the mean ± SD. Student’s *t* test was used to compare the differences between groups, followed by Bonferroni’s test. ∗∗∗∗p < 0.0001 versus control group. (F–I) Observation of the cell morphology of AT-II-S and Beas-2B-S cells by scanning electron microscopy (SEM) compared with the untransfected groups. Yellow arrows indicate the apoptotic bodies in F and G, whereas red arrows indicate the pyroptosis pores in F and I. The scale bar is indicated in the figures.

The receptor-binding domain (RBD) in the spike protein of SARS-CoV-2 is essential to bind with ACE2, and an RBD-Fc-based COVID-19 vaccine protected human ACE2 transgenic mice from SARS-CoV-2 infection (Liu et al., 2020). Therefore, to build COVID-19-infected cell models, we tried to apply spike RBD-Fc recombinant protein to AT-II cells and Beas-2B cells. Unfortunately, we did not observe any detectable changes in the growth of either cell type by CCK-8 (Figures S1A and S1B), even though we tried different dosages: 10 ng/mL, 100 ng/mL, and 5 μg/mL. Then, we tried to transfect the cells with a plasmid (NFPS-P10032 pCDNA3.4-S) containing the spike protein and constructed stable cell lines (Figure 1A). We measured the effect of the SARS-CoV-2 spike glycoprotein on AT-II cells and Beas-2B cells by CCK-8. The results showed that 4 h after planking, the cells began to adhere and grew at different speeds. Naturally, the growth of AT-II cells was much faster than the growth of Beas-2B cells (Figures 1B and 1C). SARS-CoV-2 spike glycoprotein-transfected cells demonstrated smaller cell numbers than the untransfected group (Figures 1B and 1C), which suggested that the SARS-CoV-2 spike protein hindered cell growth in both AT-II and Beas-2B cells.

We continued to detect apoptosis by flow cytometry using Annexin V/7AAD and found that in both cell lines, the spike glycoprotein of SARS-CoV-2 aggravated the ratio of apoptosis, whereas the SARS-CoV-2 spike protein induced both early apoptosis (Q3) and late apoptosis (Q3) in AT-II cells and mainly triggered late apoptosis (Q2) in Beas-2B cells (Figures 1D and 1E). In addition, necrosis was slightly induced by spike protein transfection. In AT-II-S cells, the ratio of necrosis was increased by 0.24% compared with that in AT-II cells, and Beas-2B-S cells showed a 0.14% rise compared with Beas-2B cells (Q1 of Figures 1D and 1E). Next, we observed the cell structure by using scanning electron microscopy (SEM) and found that the apoptotic bodies were released in both spike protein-transfected AT-II and Beas-2B cells (Figures 1F and 1G), which is consistent with previous reports that SARS-CoV-2 disrupts nuclear integrity and causes tissue damage (Ackermann et al., 2020; Hekman et al., 2020).

Surprisingly, SEM results also showed that some AT-II-S and Beas-2B-S cells with pores were characteristic markers of pyroptosis, whereas there were no pores in the control group cells (Figures 1H and 1I). These results are consistent with the finding that COVID-19 induces pyroptosis, which leads to the appearance of many pores on the cytoplasmic membrane and then the release of cellular content (Aglietti et al., 2016; Ma et al., 2021). Collectively, these results indicate that the spike glycoprotein of SARS-CoV-2-transfected AT-II-S and Beas-2B-S cells resembled the COVID-19-induced pneumonia and was suitable for the following study.

### SARS-CoV-2 spike protein activates pyroptosis and the inflammatory response in both AT-II cells and Beas-2B cells

**Figure 2:**
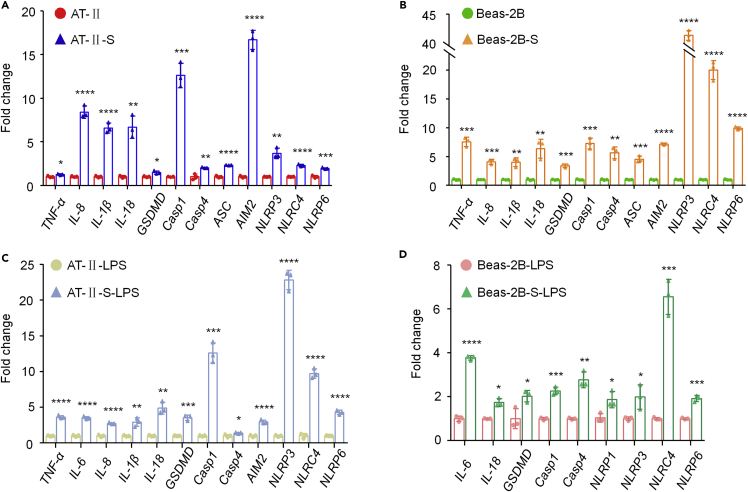
SARS-CoV-2 spike protein activates pyroptosis and the inflammatory response in both AT-II cells and Beas-2B cells and LPS treatment augments the responses (A and B) qRT–PCR results showed that the expression of inflammatory factors *TNFα*, *IL-8*, *IL-1β*, and *IL-18* and pyroptosis members *GSDMD*, *Casp1*, *Casp4*, ASC, *AIM2*, *NLRP3*, *NLRC4*, and *NLRP6* were increased in AT-II-S cells and Beas-2B-S cells, respectively, compared with that in their corresponding cells. The relative expression of target genes was normalized to 18S rRNA. The results are presented as the mean ± SD. Student’s *t* test was used to compare the differences between groups, followed by Bonferroni’s test. ∗p < 0.05, ∗∗p < 0.01, ∗∗∗p < 0.001, ∗∗∗∗p < 0.0001 versus the control group. (C) qRT–PCR results showed that the inflammatory factors *TNFα*, *IL-6*, *IL-8*, *IL-1β*, and *IL-18* and pyroptosis members *GSDMD*, *Casp1*, *Casp4*, *AIM2*, *NLRP3*, *NLRC4*, and *NLRP6* were upregulated in LPS-treated AT-II-S cells compared with LPS-treated AT-II cells. The relative expression of target genes was normalized to 18S rRNA. The results are presented as the mean ± SD. Student’s *t* test was used to compare the differences between groups, followed by Bonferroni’s test. ∗p < 0.05, ∗∗p < 0.01, ∗∗∗p < 0.001, ∗∗∗∗p < 0.0001 versus the control group. (D) qRT–PCR results showed that the inflammatory factors *IL-6*, *IL-1β*, and *IL-18* and pyroptosis members *GSDMD*, *Casp1*, *Casp4*, *NLRP1*, *NLRP3*, *NLRC4*, and *NLRP6* were increased in LPS-treated Beas-2B-S cells compared with LPS-treated Beas-2B cells. The relative expression of target genes was normalized to 18S rRNA. The results are presented as the mean ± SD. Student’s *t* test was used to compare the differences between groups, followed by Bonferroni’s test. ∗p < 0.05, ∗∗p < 0.01, ∗∗∗p < 0.001, ∗∗∗∗p < 0.0001 versus the control group.

Although some researchers have proposed targeting inflammasomes and pyroptosis for COVID-19 patient treatment (Freeman and Swartz, 2020; Yap et al., 2020), few efforts have been put into clinical trials. To confirm the profile of the inflammatory response and pyroptosis, we performed qRT–PCR and checked the transcript levels of some main characteristics (all the primers see Table 1). The results demonstrated that in AT-II cells and Beas-2B cells stably transfected with the SARS-CoV-2 spike protein, most of the tested genes were upregulated compared with the untransfected cells. In AT-II-S cells, the inflammatory factor *TNF-α* was significantly increased while *IL-8*, *IL-1β*, and *IL-18* were dramatically increased (Figure 2A). Then, we determined the expression levels of pyroptosis main factors to confirm the morphological changes in AT-II-S and Beas-2B-S cells. *Gasdermin D (GSDMD)* is an essential factor for pore formation, rupture of the cytoplasmic membrane, and leakage of the cytoplasm (Aglietti et al., 2016; Ma et al., 2021). We found that *GSDMD* was increased in AT-II-S cells (Figure 2A) and its upstream genes, *Caspase-1* (*Casp1*) and *Caspase-4* (*Casp4*), were significantly upregulated (Figure 2A). The following upstream genes of inflammasomes, *ASC*, *AIM2*, *NLRP3*, *NLRC4*, and *NLRP6*, were also increased by SARS-CoV-2 spike protein transfection in AT-II cells (Figure 2A). Similar phenomena were detected in Beas-2B-S cells. We found that four inflammatory factors, *TNF-α*, *IL-8*, *IL-1β*, and *IL-18*, were all significantly increased (Figure 2B). The altered pyroptosis main factors in AT-II-S cells were also increased in Beas-2B-S cells (Figure 2B). In particular, *NLRP3* and *NLRC4* were upregulated more than 35-fold (Figure 2B). All these results confirmed that the spike protein of SARS-CoV-2 induced inflammatory responses and pyroptosis in both AT-II cells and Beas-2B cells.

**Table 1:** Primers that were used in this study

Gene name	Primer name	Primer sequence (5' to 3′)
*18S ribosomal RNA (18S)*	Hsp-18s-RTF	CAGCCACCCGAGATTGAGCA
	Hsp-18s-RTR	TAGTAGCGACGGGCGGTGTG
*interleukin 8 (IL-8)*	Hsp-IL-8-RTF1	CTCTCTTGGCAGCCTTCCT
	Hsp-IL-8-RTR1	AAATTTGGGGTGGAAAGGTT
*interleukin 18 (IL-18)*	Hsp-IL-18-RTF	TGGCTGCTGAACCAGTAGAA
	Hsp-IL-18-RTR	TCAAATAGAGGCCGATTTCC
*interleukin 1 beta (IL-1β)*	Hsp-IL-1β-RTF	GACAAAATACCTGTGGCCTT
	Hsp-IL-1β-RTR	ATCTACACTCTCCAGCTGTA
*gasdermin D (GSDMD)*	Hsp-*GSDMD*–RTF	GACCCTAACACCTGGCAGAC
	Hsp-*GSDMD*–RTR	GTCTGCAGCACCTCAGTCAC
*tumor necrosis factor alpha-like (TNF-α)*	Hsp-TNF-α-RTF	GATCATCTTCTCGAACCCC
	Hsp-TNF-α-RTR	AAGAGGACCTGGGAGTAGAT
*Caspase-1 (Casp1)*	Hsp-Casp1-RTF	TGGGACTCTCAGCAGATCAA
	Hsp-Casp1-RTR	TGGGACTCTCAGCAGATCAA
*Caspase-4 (Casp4)*	Hsp-Casp4-RTF	CGGGTCATGGCAGACTCTA
	Hsp-Casp4-RTR	GACAAAGCTTGAGGGCATCT
*NLR family*, *pyrin domain containing 1 (NLRP1)*	Hsp-NLRP1–RTF	GGACCTAGCCCTCCATACCT
	Hsp-NLRP1–RTR	TCAGGCAGCTGTCTCAAAAC
*NLR family*, *pyrin domain containing 3 (NLRP3)*	Hsp-NLRP3–RTF	AGCCACGCTAATGATCGACT
	Hsp-NLRP3–RTR	AACCCATCCACTCCTCTTCA
*NLR family*, *CARD domain containing 4 (NLRC4)*	Hsp-NLRC4-RTF	CAGCCTGTTGAAACATTTGG
	Hsp-NLRC4-RTR	ATACACCCATGAAGGCAAGC
*NLR family*, *pyrin domain containing 6 (NLRP6)*	Hsp-NLRP6–RTF	CTGAGCTACTGCGTGAGGTG
	Hsp-NLRP6–RTR	TTGTTTTGTGGTGCCTTGAG
*absent in melanoma 2 (AIM2)*	Hsp-AIM2–RTF	GCAGTGATGAAGACCATTCG
	Hsp-AIM2–RTR	GCTTTGCGACATCATTTCTG
*Apoptosis associated speck like protein containing a CARD (ASC)*	Hsp-ASC-RTF	AGCCAGGCCTGCACTTTAT
	Hsp-ASC-RTR	CTGGTACTGCTCATCCGTCA
*angiotensin converting enzyme 2 (ACE2)*	Hsp-ACE2-MA-s1	GGACAAGTTTAACCACGAAGCC
	Hsp-ACE2-MA-as1	CAGCTGAAGCTTGACTGTGAGAT
*angiotensin converting enzyme 2 (ACE2)*	Hsp-ACE2-RTF	TTAACCACGAAGCCGAAGAC
	Hsp-ACE2-RTR	TACATTTGGGCAAGTGTGGA
*SARS-COV-2-Spike (C0V-2-S)*	C0V-2-S-RTF5	AGACCCAGAGCCTGCTGATA
	C0V-2-S-RTR5	GCTCTCCATCCAGCTCTTGT
*interferon gamma (INF-γ)*	Mus-INF-γ-RTF	GCCATCAGCAACAACATAAG
	Mus-INF-γ-RTR	GGGTTGTTGACCTCAAACTT
*interleukin 4 (IL-4)*	Mus-IL-4-RTF	CAGTTCTACAGCCACCATGA
	Mus-IL-4-RTR	TACTCTGGTTGGCTTCCTTC
*interleukin 6 (IL-6)*	Mus-IL-6-RTF	AGAGGATACCACTCCCAACA
	Mus-IL-6-RTR	CAGTTTGGTAGCATCCATCA
*interleukin 10 (IL-10)*	Mus-IL-10-RTF	CCTTATCGGAAATGATCCAG
	Mus-IL-10-RTR	CTCCACTGCCTTGCTCTTAT
*interleukin 12 (IL-12)*	Mus-IL-12-RTF	ACACTGGACCAAAGGGACTA
	Mus-IL-12-RTR	AAGGCTTCATCTGCAAGTTC
*interleukin 13 (IL-13)*	Mus-IL-13-RTF	GGAGCTTATTGAGGAGCTGA
	Mus-IL-13-RTR	AGGTCCACACTCCATACCAT
*interleukin 15 (IL-15)*	Mus-IL-15-RTF	AGAAACGTGCTCTACCTTGC
	Mus-IL-15-RTR	GAACATTTGGACAATGCGTA
*interleukin 17 (IL-17)*	Mus-IL-17-RTF	GACTACCTCAACCGTTCCAC
	Mus-IL-17-RTR	TCTTGCTGGATGAGAACAGA
*interleukin 18 (IL-18)*	Mus-IL-18-RTF	TCTTGCGTCAACTTCAAGGA
	Mus-IL-18-RTR	GGCCAAAGTTGTCTGATTCC
*interleukin 1 beta (IL-1β)*	Mus-IL-1β-RTF	TGTGGCAGCTACCTGTGTCT
	Mus-IL-1β-RTR	TCCATTGAGGTGGAGAGCTT
*C-X-C motif chemokine ligand 10 (CXCL10)*	Mus-CXCL10-RTF	AGTGAGAATGAGGGCCATAG
	Mus-CXCL10-RTR	CTTAGATTCCGGATTCAGACA
*C-C motif chemokine ligand 2 (CCL2)*	Mus-CCL2-RTF	GATGATCCCAATGAGTAGGC
	Mus-CCL2-RTR	TCTCTTGAGCTTGGTGACAA
*Caspase-1 (Casp1)*	Mus-Casp1-RTF	GGAGCTTCAATCAGCTCCAT
	Mus-Casp1-RTR	CTTGAGGGTCCCAGTCAGTC
*Caspase-4 (Casp4)*	Mus-Casp4-RTF	TGGTGGTGAAAGAGGAGCTT
	Mus-Casp4-RTR	GCCATGAGACATTAGCACCA
*gasdermin D (GSDMD)*	Mus-GSDMD–RTF	AGTGCTCCAGAACCAGAACC
	Mus-GSDMD–RTR	ATTCATGGAGGCACTGGAAC
*NLR family*, *pyrin domain containing 1 (NLRP1)*	Mus-NLRP1–RTF	TCATGGTGGTCACTTTCTGC
	Mus-NLRP1 -RTR	GGATTTCCACTGAGGTCCAA
*NLR family*, *pyrin domain containing 3 (NLRP3)*	Mus-NLRP3–RTF	AAGAAGGACCAGCCAGAGTG
	Mus-NLRP3–RTR	ATGGAGATGCGGGAGAGATA
*NLR family*, *CARD domain containing 4 (NLRC4)*	Mus-NLRC4-RTF	GGCCTGCAACCTCTTTCTTA
	Mus-NLRC4-RTR	CGATGGTCCTTCTTCCACAT
*NLR family*, *pyrin domain containing 6 (NLRP6)*	Mus-NLRP6–RTF	CCCCGAAATGTCATCTGAGT
	Mus-NLRP6–RTR	CCCCGAAATGTCATCTGAGT
*absent in melanoma 2 (AIM2)*	Mus-AIM2–RTF	CCTGATTCAAAGTGCAGGTG
	Mus-AIM2–RTR	GCAGAGCAGTTTTCAGCTTG
*Apoptosis associated speck like protein containing a CARD (ASC)*	Mus-ASC-RTF	GAGCAGCTGCAAACGACTAA
	Mus-ASC-RTR	CACTCCGTCCACTTCTGTGA
*tumor necrosis factor alpha-like (TNF-α)*	Mus-TNF-α-RTF	GAACTGGCAGAAGAGGCACT
	Mus-TNF-α-RTR	AGGGTCTGGGCCATAGAACT

### LPS treatment aggravates the inflammatory response and pyroptosis in SARS-CoV-2 spike protein-transfected AT-II and Beas-2B cells

To test whether SARS-CoV-2 spike protein-transfected AT-II and Beas-2B cells would be much more sensitive to the inflammatory response and pyroptosis than normal cells, we then treated the cells with lipopolysaccharide (LPS), which is known to trigger pyroptosis and inflammation (Brooks et al., 2020; Rathinam et al., 2019). Our results demonstrated that in LPS-treated AT-II-S cells, five inflammatory factors, *TNF-α*, *IL-6*, *IL-8*, *IL-1β* and *IL-18*, were increased compared with those in LPS-treated AT-II cells (Figure 2C). Although *IL-6* was not changed in AT-II cells compared with AT-II cells (Figure S2A), LPS treatment activated *IL-6* mRNA expression in AT-II-S cells (Figure 2C), whereas *ASCs* were upregulated by spike protein transfection in AT-II cells (Figure 2A) but inactivated by LPS treatment (Figure S2C). LPS treatment approximately increased *NLRP3* by 23-fold, whereas only a 3.5-fold increase was observed without LPS in AT-II-S cells compared to that in the control AT-II cells (Figure 2C). *TNF-α*, *IL-8*, *ASC*, and *AIM2* were significantly upregulated in Beas-2B-S cells compared to Beas-2B cells (Figure 2D), but we did not detect any dramatic alterations in these genes in the LPS treatment groups (Figure S2D). Although *IL-1β*, *IL-18*, *GSDMD*, *NLRP3*, *NLRC4*, and *NLRP6* were also increased by LPS treatment in Beas-2B-S cells compared to Beas-2B cells, their change folds were within a narrower range than that in the non-LPS treatment group (Figure 2D). We also found that LPS significantly increased the transcript expression levels of *IL-6* and *NLRP1* in Beas-2B-S cells (Figure 2D), but no detectable change was observed in Beas-2B cells compared with their relative control group (Figure S2B). These results suggested that LPS treatment could aggravate the inflammatory response and pyroptosis in SARS-CoV-2 spike protein-transfected AT-II and Beas-2B cells compared to their corresponding untransfected cells. They also indicated that the two different types of lung cells have different reactions to spike protein transfection and LPS stimulation.

### The main factors of the inflammatory response and pyroptosis were downregulated in LPS-treated AT-II and Beas-2B cells by ACE2-MSCs application

**Figure 3:**
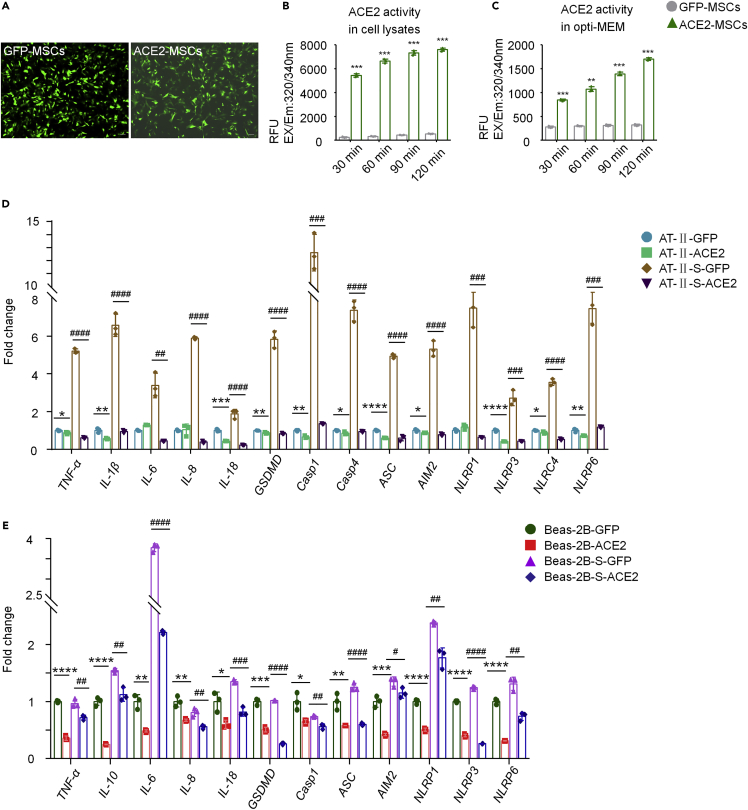
The main factors of the inflammatory response and pyroptosis were downregulated in LPS-treated AT-II and Beas-2B cells by ACE2-MSCs application (A) GFP-MSCs and ACE2-MSCs were detected by fluorescence microscopy. Scale bar: 50 μm. (B and C) ACE2 activity in cell lysates and cell culture medium was measured by an ACE2 activity assay kit, respectively. The results are presented as the mean ± SD. Student’s *t* test was used to compare the differences between groups, followed by Bonferroni’s test. ∗∗p < 0.01, ∗∗∗p < 0.001, versus control group. (D) qRT–PCR results of the effects of ACE2-MSCs and GFP-MSCs on LPS-treated AT-II and AT-II-S cells. The inflammatory factors *TNFα*, *IL-1β*, *IL-6*, and *IL-8* were decreased in LPS-treated AT-II/S cells cocultured with ACE2-MSCs compared with the control group cocultured with GFP-MSCs. *GSDMD*, *Casp1* and *Casp4*, *ASC*, *AIM2*, *NLRP1*, *NLRP3*, *NLRC4*, and *NLRP6* were downregulated in LPS-treated AT-II/-S cells cocultured with ACE2-MSCs compared with the control group cocultured with GFP-MSCs. The relative expression of target genes was normalized to 18S rRNA. The results are presented as the mean ± SD. Student’s *t*-test was used to compare the differences between groups, followed by Bonferroni’s test. ∗p < 0.05, ∗∗p < 0.01, ∗∗∗p < 0.001, ∗∗∗∗p < 0.0001 versus the control group. (E) qRT–PCR results of the effects of ACE2-MSCs and GFP-MSCs on LPS-treated Beas-2B and Beas-2B-S cells. The transcript levels of *TNFα*, *IL-10*, *IL-6*, *IL-8*, *GSDMD*, *Casp1*, *ASC*, *AIM2*, *NLRP1*, *NLRP3*, and *NLRP6* were repressed in LPS-treated Beas-2B-S cells cocultured with ACE2-MSCs compared with the control group cocultured with GFP-MSCs. The relative expression of target genes was normalized to 18S rRNA. The results are presented as the mean ± SD. Student’s *t* test was used to compare the differences between groups, followed by Bonferroni’s test. ∗p < 0.05, ∗∗p < 0.01, ∗∗∗p < 0.001, ∗∗∗∗p < 0.0001 versus the control group.

To examine the therapeutic effects of ACE2-MSCs on COVID-19, we overexpressed ACE2 in hUC-MSCs (ACE2-MSCs) and used the empty vector as a control (GFP-MSCs) (Figure 3A). ACE2 activities in cell lysates and culture medium were measured, and the results indicated that ACE2 activities were increased with culture time in ACE2-MSCs (Figures 3B and 3C), which suggested that hUC-MSCs successfully overexpressed ACE2 and would be effective for subsequent research. We treated AT-II, AT-II-S, Beas-2B, and Beas-2B-S cells with LPS for 24 h and then cocultured them with ACE2-MSCs or GFP-MSCs via a Transwell coculture system. The transcript levels of the main factors of inflammation and pyroptosis were examined by qRT–PCR (all the primers see Table 1), and the results showed that ACE2-MSCs very obviously inhibited all these factors in LPS-treated AT-II-S cells compared with the GFP-MSCs treatment group (Figure 3D). Surprisingly, in LPS-treated AT-II cells, some genes, such as *IL-6*, *IL-8*, and *NLRP6*, were not changed, whereas other pyroptosis main factors, such as *GSDMD*, *Casp1*, *Casp4*, *ASC*, *AIM2*, *NLRP1*, and *NLRC4*, were obviously repressed (Figure 3D). However, in LPS-treated Beas-2B cells, regardless of whether they were transfected with the spike protein, ACE2-MSCs significantly restrained the expression of almost all the tested genes (Figure 3E), except *IL-1β*, *Casp4*, and *NLRC4*. These results probably indicate that ACE2-MSCs function in different ways in two different types of lung cells but have notable effects on inhibiting inflammation and pyroptosis induced by spike protein transfection and LPS treatment compared with the administration of GFP-MSCs.

### SARS-CoV-2 pseudovirus deteriorates mouse lung injury induced by LPS and increases the transcript expression levels of the main factors of cytokine storm and pyroptosis

**Figure 4:**
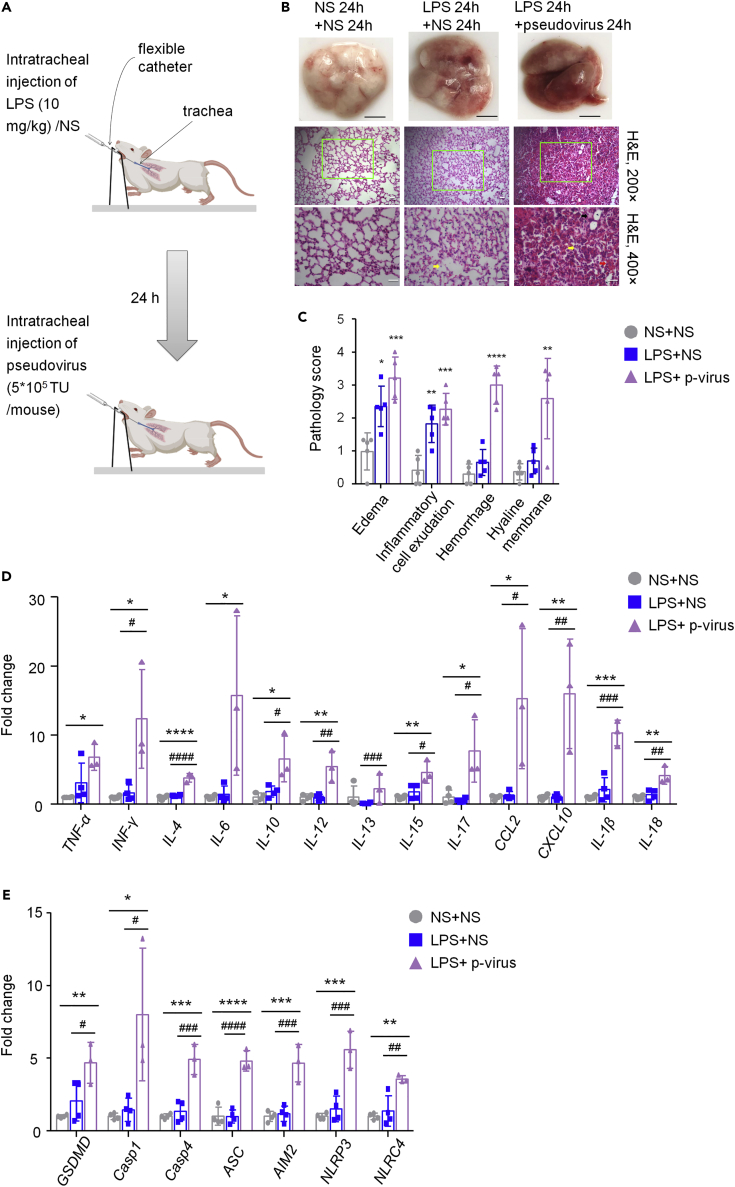
SARS-CoV-2 pseudovirus deteriorates mouse lung injury induced by LPS and increases the transcript expression levels of the main factors of cytokine storm and pyroptosis (A) Experimental scheme for the SARS-CoV-2 pseudovirus-infected mouse model. Twenty-four hours after intratracheal injection of LPS (10 mg/kg) or normal saline (NS), 5∗10^5^ TU SARS-CoV-2 pseudovirus or saline was injected in the same way. (B) SARS-CoV-2 pseudovirus deteriorates mouse lung injury in LPS-induced ALI. Gross pathology and histopathology of lung tissues from three groups: NS 24 h + NS 24 h, LPS 24 h + NS 24 h, and LPS 24 h + SARS-CoV-2 pseudovirus 24 h were shown. The yellow arrow indicates the exudation of inflammatory cells, the black arrow indicates the hyaline membrane, and the red arrow indicates hemorrhage. Scale bars: white, 0.5 cm; black, 50 μm. (C) Pathology scores of edema, inflammatory cell exudation and hemorrhage, and hyaline membrane. The results are presented as the mean ± SD. One-way ANOVA was used to compare the differences between groups, followed by Bonferroni’s test. N = 5 per group, ∗p < 0.05, ∗∗p < 0.01, ∗∗∗p < 0.001, ∗∗∗∗p < 0.0001 versus the NS 24 h + NS 24 h group. (D) qRT–PCR was used to examine the transcript levels of core inflammatory factors of cytokine storms: *TNFα*, *INF-γ*, *IL-4*, *IL-6*, *IL-10*, *IL-12*, *IL-13*, *IL-15*, *IL-17*, *CCL2*, *CXCL10*, *IL-1β*, and *IL-18*. The relative expression of target genes was normalized to 18S rRNA. The results are presented as the mean ± SD. *t*-test or one-way ANOVA was used to compare the differences between groups, followed by Bonferroni’s test. N = 3 or 4 per group, ∗p < 0.05, ∗∗p < 0.01, ∗∗∗p < 0.001, ∗∗∗∗p < 0.0001 versus the NS 24 h + NS 24 h group. (E) qRT–PCR detected the main pyroptosis factor mRNAs: *GSDMD*, *Casp1*, *Casp4*, *ASC*, *AIM2*, *NLRP3*, and *NLRC4*. The relative expression of target genes was normalized to 18S rRNA. The results are presented as the mean ± SD. One-way ANOVA was used to compare the differences between groups, followed by Bonferroni’s test. N = 3 or 4 per group, ∗p < 0.05, ∗∗p < 0.01, ∗∗∗p < 0.001, ∗∗∗∗p < 0.0001 versus the NS 24 h + NS 24 h group.

*In vitro*, we proved that the spike protein of SARS-CoV-2 transfection similar to the phenomena (pyroptosis, apoptosis, and inflammation) caused by SARS-CoV-2 virus infection and that the administration of ACE2-MSCs suppressed the main factors of pyroptosis and inflammation. Next, we aimed to confirm whether ACE2-MSCs are much more effective in COVID-19 treatment than MSCs alone *in vivo*. Considering the biosafety and operability to construct a SARS-CoV-2 virus-infected mouse model, we preferred to use the SARS-CoV-2 pseudovirus for infection. Pseudoviruses expressing the SARS-CoV-2 spike protein that could simulate the real virus to infect target cells without replication have been used widely as an alternative strategy for studying COVID-19 treatment (Liu et al., 2020; Xia et al., 2020a, 2020b). hACE2 transgenic mouse models are valuable for understanding the pathogenesis of COVID-19, as well as designing therapeutic strategies (Bao et al., 2020; Jiang et al., 2020; Winkler et al., 2020; Zheng et al., 2021). Here, we performed intratracheal injection of SARS-CoV-2 pseudovirus in hACE2 transgenic mice to mirror COVID-19. The experimental strategy is shown in Figure 4A. Unfortunately, if we only intratracheally injected the pseudovirus for 24 h, we could not observe detectable histopathological changes (Figure S3). However, based on LPS-induced ALI, intratracheal injection of SARS-CoV-2 pseudovirus deteriorated lung injury (Figure 4B). The results of gross pathology and histopathology of lungs from injecting the saline twice (sham group), LPS injection plus saline (control group), and LPS injection plus SARS-CoV-2 pseudovirus injection showed that LPS treatment induced the lung injury, and the pseudovirus made the lung injury worse: much more edema, inflammatory cell exudation, hemorrhage, and hyaline membrane were detected (Figures 4B and 4C); however,gender has no detectable effect on the lung injury. To clearly understand how the SARS-CoV-2 pseudovirus attacks lung tissues, we checked various factors released during cytokine storm and noted that *TNF-α*, *INF-γ*, *IL-4*, *IL-6*, *IL-10*, *IL-12*, *IL-13*, *IL-15*, *IL-17*, *CCL2*, and *CXCL10* were all activated by LPS treatment and much more upregulated by SARS-CoV-2 pseudovirus infection (Figure 4D), which suggested that the SARS-CoV-2 pseudovirus triggered cytokine storm (Liu et al., 2021; Su et al., 2020). The mRNA levels of pyroptosis main factors were then determined. Notably, in addition to *IL-1β*, the transcript of *IL-18*, another downstream marker of pyroptosis,was dramatically promoted in the SARS-CoV-2 pseudovirus-infected mice compared to both the control and sham groups (Figure 4E). Following their upstream gene *GSDMD*, its mRNA level was indisputably increased in the SARS-CoV-2 pseudovirus-infected mice (Figure 4E). Interestingly, both *Casp1* and *Casp4* were aggravated by the SARS-CoV-2 pseudovirus (Figure 4E). We also found that the transcript levels of inflammasomes, including *ASC*, *AIM2*, *NLRP3*, and *NLRC4* were increased to different extents (Figure 4E), suggesting that pyroptosis was stirred up by SARS-CoV-2 pseudovirus infection. Taken together, these results implied that our COVID-19-infected mouse model was valuable for subsequent studies.

### ACE2-MSCs alleviate SARS-CoV-2 pseudovirus-induced lung injury and inhibit the main factors mRNA of cytokine storm and pyroptosis

**Figure 5:**
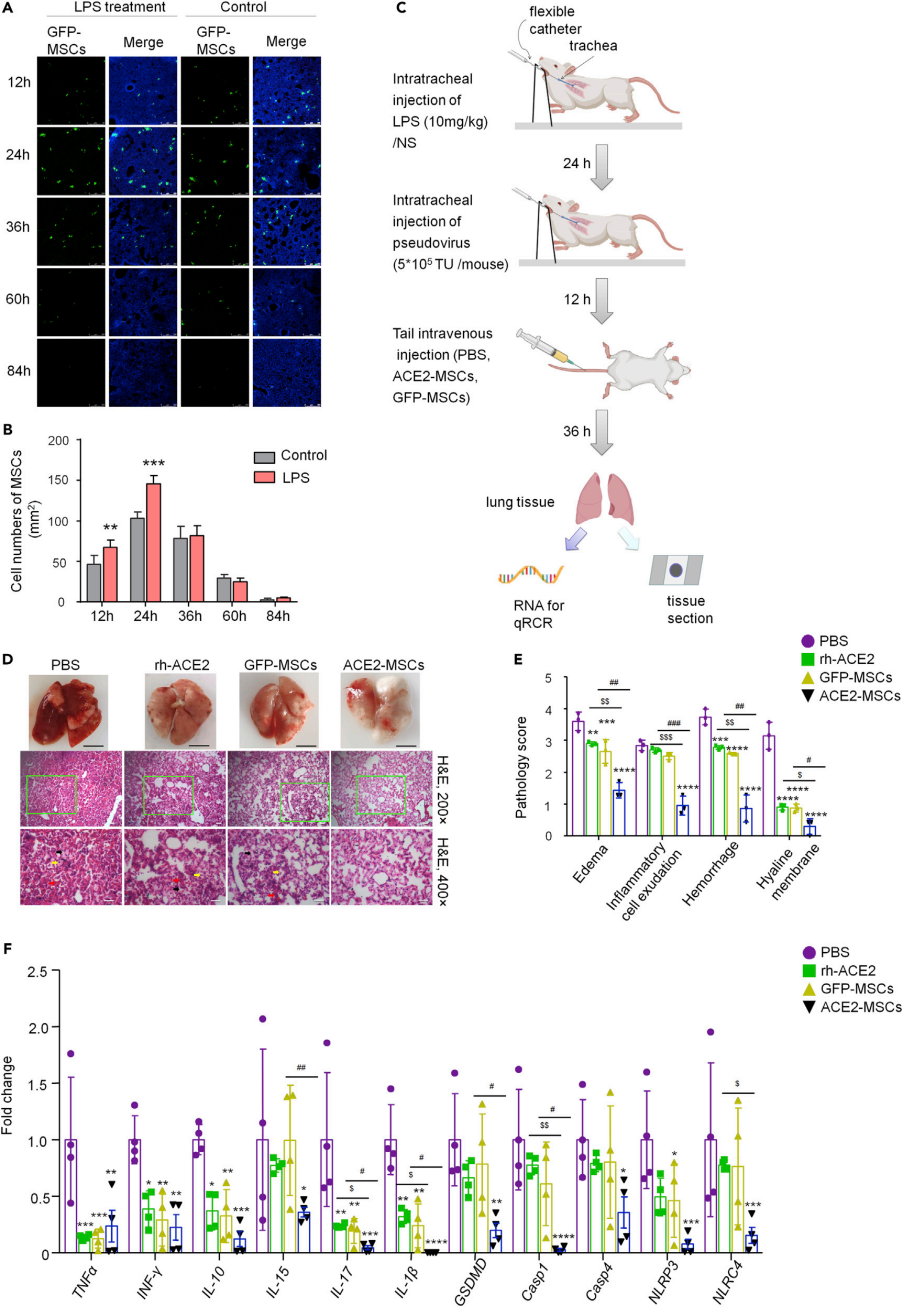
ACE2-MSCs alleviate SARS-CoV-2 pseudovirus-induced lung injury and inhibit the main factors mRNA of cytokine storm and pyroptosis (A) Tracing GFP-MSCs distribution in injured lungs and normal lungs. GFP+ cells were detected by fluorescence microscopy after tail vein injection in LPS-treated mouse lungs and normal mouse lungs. Scale bar: 250 μm. (B) The cell numbers of GFP-MSCs per mm^2^ lung tissue were counted after tail vein injection. The results are presented as the mean ± SD. Student’s *t*-test was used to compare the differences between groups, followed by Bonferroni’s test. N = 6 per group, ∗∗p < 0.01, ∗∗∗p < 0.001 verse control group. (C) Experimental scheme MSCs treatment. Twenty-four hours after intratracheal injection of LPS (10 mg/kg) or saline (NS), 5∗10^5^ TU SARS-CoV-2 pseudovirus or saline was injected, 12 h later followed by tail intravenous injection of PBS, ACE2-MSCs, or GFP-MSCs. (D) Histological evaluation of the therapeutic potential of rh-ACE2, ACE2-MSCs, and GFP-MSCs in SARS-CoV-2 pseudovirus-induced lung injury. Gross pathology and histopathology of lung tissues from three treatment groups: PBS, rh-ACE2, GFP-MSCs, and ACE2-MSCs are shown. Tail vein injection of PBS was the control group. The yellow arrow indicates the exudation of inflammatory cells, the black arrow indicates the hyaline membrane, and the red arrow indicates hemorrhage. Scale bars: white, 0.5 cm; black, 50 μm. (E) Pathology scores of edema, inflammatory cell exudation, hemorrhage, and hyaline membrane. The results are presented as the mean ± SD. Two-way ANOVA was used to compare the differences between groups, followed by Bonferroni’s test. N = 3 per group, ∗∗∗p < 0.001, ∗∗∗∗p < 0.0001 versus the PBS treatment group; ^##^p < 0.01, ^###^p < 0.001 indicated GFP-MSCs treatment group versus the ACE2-MSCs treatment group; ^$$^p < 0.01, ^$$$^p < 0.001 indicated rh-ACE2 treatment group versus the ACE2-MSCs treatment group. (F) qRT–PCR results of the main factors of cytokine storm and pyroptosis core members that were inhibited by ACE2-MSCs and GFP-MSCs injection. The relative expression of target genes was normalized to 18S rRNA. The results are presented as the mean ± SD. Two-way ANOVA was used to compare the differences between groups, followed by Bonferroni’s test. N = 4 per group, ∗p < 0.05, ∗∗p < 0.01, ∗∗∗p < 0.001 versus the PBS treatment group. ^#^p < 0.05, ^##^p < 0.01 indicated GFP-MSCs treatment group versus the ACE2-MSCs treatment group; ^$^p < 0.05, ^$$^p < 0.01 indicated rh-ACE2 treatment group versus the ACE2-MSCs treatment group.

To ensure that tail intravenous injection of MSCs homed to the lung tissues to exert their functions, we traced the distribution of MSCs from 12 h to 84 h after injection. The results demonstrated that in the LPS-treated group at every tested time point, the number of MSCs was greater than that in the control group, and at the peak time of accumulation (24 h), the difference was obvious (Figures 5A and 5B). Considering the accumulation and actual action of MSCs on lung tissues, we treated lung injury with MSCs for 36 h and sampled lung tissues for the following experiments (Figure 5C). Figure 5D shows the histopathology of PBS-treated, GFP-MSCs-treated, or ACE2-MSCs-treated injured lung tissues. Compared with the PBS-treated group, the GFP-MSCs group had significantly reduced lung injury, but the ACE2-MSCs administration group showed much more significant therapeutic effects (Figures 5D and 5E). The results of gross histopathology of the lungs showed that GFP-MSCs administration significantly reduced the hemorrhage positions, whereas the application of ACE2-MSCs aided with a near-normal recovery of the damaged lung tissue (Figure 5D). Histopathological examination by hematoxylin-eosin staining showed that inflammatory cell exudation, hemorrhage, and hyaline membrane were barely detected in the ACE2-MSCs treatment group (Figure 5D). Statistical analysis of the histopathological scores found that edema, inflammatory cell exudation, hemorrhage, and hyaline membrane were all decreased by the administration of GFP-MSCs and ACE2-MSCs to different degrees (Figure 5E). Again, ACE2-MSCs functioned much more effectively than MSCs alone to treat COVID-19-induced lung injuries. Besides, we compared the therapeutics of soluble ACE2 and the therapeutics of ACE2-MSCs. When compared with the control group, human ACE-2 (rh-ACE2) protein could attenuate the lung injury; however, its therapeutic effects were weaker than that of ACE2-MSCs in COVID-19 treatment (Figures 5D and 5E).

To explore the mechanisms underlying the therapeutic effect, we tested the expression levels of the mentioned inflammatory factors and pyroptosis factors induced by LPS treatment and SARS-CoV-2 pseudovirus infection. Even though 13 core members of the cytokine storm and 7 vital factors of pyroptosis were triggered in the SARS-CoV-2 pseudovirus-infected hACE2 transgenic mouse model, only 6 core members of the cytokine storm (*TNF-α*, *INF-γ*, *IL-10*, *IL-15*, *IL-17*, and *IL-1β*) and 5 vital factors of pyroptosis (*GSDMD*, *Casp1*, *Casp4*, *NLRP3*, and *NLRC4*) were repressed by ACE2-MSCs and GFP-MSCs (Figure 5F), whereas other genes were not altered by the administration of ACE2-MSCs or GFP-MSCs (Figure S4). Our results also showed that ACE2-MSCs much more strongly inhibited the expression levels of *IL-15*, *IL-17*, *IL-1β*, *GSDMD*, and *Casp1* than MSCs alone (Figure 5F). Using rh-ACE2, only the inflammatory factors such as *TNF-α*, *INF-γ*, *IL-10*, *IL-17*, and *IL-1β* were significantly repressed (Figure 5F), which indicated that the therapeutic effects of ACE2-MSCs were much stronger than the therapeutic effects of rh-ACE2. In summary, our study proved that the administration of MSCs could alleviate COVID-19-induced lung injury and repress cytokine storms and pyroptosis at the molecular level. In addition, ACE2-MSCs function more effectively than GFP-MSCs or rh-ACE2.

## Discussion

Defeating COVID-19 is still an emergency, especially after the mutated virus has appeared and as it continues to ravage the whole world. Recently, most of the focus has been on exploring vaccines and some vaccines have targeted the SARS-CoV-2 spike protein (Keech et al., 2020; Walsh et al., 2020). Even though vaccines are immensely helpful in reducing the infection rate and also in alleviating the symptoms of COVID-19 in patients who were infected despite being vaccinated, the deviation off-target of vaccines should not be neglected. Therefore, numerous preclinical and clinical studies have insisted on sticking to COVID-19 patient treatment, targeting cytokine storms, pyroptosis, and other factors that lead to death (Diorio et al., 2020; Karki et al., 2021; Pan et al., 2021a; Shi et al., 2021). Here, we aimed to determine some general solution to alleviate COVID-19-induced lung injury. To mirror the real SARS-CoV-2 virus infection course, *in vitro*, we selected two main lung cell types, AT-II and Beas-2B, and transfected the cells with SARS-CoV-2 spike protein containing plasmid DNA, which showed apoptosis, necrosis, and pyroptosis; *in vivo*, hACE2 transgenic mice were treated with LPS first and infected with SARS-CoV-2 pseudovirus, which demonstrated severe lung injuries, cytokine storm, and pyroptosis. The application of MSCs significantly reduced the transcript levels of the main factors of the inflammatory response, cytokine storm, and pyroptosis, especially ACE2-MSCs, which were much more effective than MSCs alone.

We tried to use different doses of SARS-CoV-2 spike RBD recombinant protein to infect AT-II and Beas-2B cells for several days but did not yield obvious results. However, when we transfected the cells with recombinant plasmid DNA containing the full-length sequence of the SARS-CoV-2 spike protein, the results were unsurprising. Cell growth of SARS-CoV-2 spike protein-transfected AT-II and Beas-2B cells was inhibited 12 h after cell adhesion and exhibited the most significant decrease at 36 h in the two cell types (Figures 1B and 1C), and AT-II-S cells showed a sharp decline. The numbers of apoptotic AT-II-S and Beas-2B-S cells were similar. Together, the results suggest that AT-II cells are more susceptible to infection by SARS-CoV-2, as confirmed by previous reports. AT-II cells abundantly express ACE2, which binds to the SARS-CoV-2 virus; after infection, AT-II cells trigger the inflammatory response, and autopsy studies of COVID-19 patients demonstrated the appearance of necrosis and cell hyperplasia of AT-II cells (Ackermann et al., 2020; Satarker et al., 2021). The SARS-CoV-2 spike protein enhanced the expression of ACE2 in Beas-2B cells, and the cytokines IL-4 and IL-13 were repressed, whreas TNF-α, IL-12, and IL-17 increased ACE2 expression in Beas-2B cells, which reflects the different risks of severe COVID-19 (Song et al., 2021; Zhou et al., 2021). None of these studies have detected the SARS-CoV-2 triggered pyroptosis phenomena yet; however,we found that besides cell growth repression, apoptosis, and necrosis, various pores which stand for pyroptosis were seen in both AT-II and Beas-2B Cells. SARS-CoV-2 pseudovirus-infected hACE2 transgenic mice did not show any significant phenotypic change. Combined with LPS treatment, SARS-CoV-2 pseudovirus-infected mice demonstrated severe lung injuries, which might be because of cytokine storms and pyroptosis. Thus, for basic medical research, pseudoviruses or recombinant virus proteins should be considered substitutes for researching real harmful or even devastating viruses.

Cell death is one of the most important factors linking cytokine storms and histopathological changes during SARS-CoV-2 infection, and several viral proteins of SARS-CoV-2 induce cell death — the so-called PANoptosis (pyroptosis, apoptosis, and necroptosis) (Frühbeck et al., 2021; Karki et al., 2021; Lee et al., 2020). Three major cell death pathways likely occur simultaneously in COVID-19 patients. Apoptosis has been suggested as the critical type of cell death in the outcome of COVID-19 and depends on the cells and the timing (Paolini et al., 2021). Necroptosis was activated by RIPK3, which was directly affected by SARS-CoV-2 virus (Li et al., 2020). Pyroptosis is well-known to be activated by inflammasome components (NLRP3, active Casp-1) and then executed by gasdermin family members. GSDMD is the most popular inflammasome mediated by Casp1 (classical pathway) or Casp4/5/11 (noncanonical pathway) and finally secretes the downstream proinflammatory mediators IL-1β and IL-18 (Kayagaki et al., 2015; Shi et al., 2015). However, the ratio of three types of cell death is unclear. Our current research mainly focused on pyroptosis and confirmed that pyroptosis was triggered by the SARS-CoV-2 spike protein in both AT-II and Beas-2B cells, not only by morphological observation but also by gene expression patterns. Cleavage of GSDMD and inflammasome components (*ASC*, *AIM2*, *NLRP3*, *NLRC4*, *NLRP6*, *Casp1*, and *Casp4*) were all activated by SARS-CoV-2 spike protein transfection; *AIM2* and *Casp1* were upregulated by more than 10-fold in AT-II-S cells, whereas *NLRP3* and *NLRC4* were increased by more than 20-fold in Beas-2B-S cells (Figures 2A and 2B). Most likely because of the numerous pores in Beas-2B-S cells, the main factors of pyroptosis were activated more strongly than those in AT-II-S cells. Almost all of the papers that studied SARS-CoV-2 induced pyroptosis give the credit to Casp1, the downstream factor of NLRP3 (Kayagaki et al., 2015; Shi et al., 2015); however, our results demonstrated that not only Casp1 but also Casp4 were stirred up by SARS-CoV-2 spike protein transfection *in vitro* and pseudovirus infection *in vivo* (Figures 2A–2D), which indicated that both recombinant SARS-CoV-2 spike protein and pseudovirus not only dominantly triggered the classical pyroptosis pathway but also induced the noncanonical pathway.

There are many preclinical and clinical trials targeting NLRP3 and GSDMD to treat COVID-19 patients (Ma et al., 2021; Pan et al., 2021b; Vora et al., 2021). In our study, we also found that these two factors were highly increased. However, we should also take notice of *AIM2* and *NLRC4* because their transcript expression was much higher upregulated by spike protein transfection in AT-II and Beas-2B cells (Figures 2A–2D) and induced by pseudovirus infection in hACE2 transgenic mice (Figure 4E), and previous studies have shown that autoinflammation with pyroptosis in a COVID-19 patien was because of mutations in NLRC4 (Duncan-Lowey et al., 2021) and COVID-19 infecting blood monocytes to activate the AIM2 inflammasome (Junqueira et al., 2021). Moreover, the inflammasome components, *ASC* and *NLRP6*, were also activated *in vitro* (Figures 2A–2D). There are many reports about the roles of NLRP1 in the lungs, but only a few reports have mentioned the functions of *ASC* and *NLRP6* in the lungs (Gao et al., 2019; Ghimire et al., 2018). Therefore, our research might expand the scope of other related research works.

“Cytokine storm” is known as the release of a large amount of proinflammatory cytokines that lead to the death of certain COVID-19 patients because it is directly related to lung injury, multiorgan failure, and other prognoses (Chen et al., 2020; Huang et al., 2020). In our COVID-19 mouse model, the analysis of cytokine levels in lung tissues revealed elevated levels of *TNF-α*, *INF-γ*, *IL-4*, *IL-6*, *IL-10*, *IL-12*, *IL-13*, *IL-15*, *IL-17*, *CCL2*, and *CXCL10* (Figure 4D), which is consistent with the results from the plasma of COVID-19 patients (Huang et al., 2020). These findings might provide an avenue to develop therapeutic approaches for preventing COVID-19-associated morbidity and mortality.

Various clinical trials have proven that hUC-MSCs treatment is a potentially effective therapeutic approach for COVID-19 patients (Dilogo et al., 2021; Kouroupis et al., 2021; Lanzoni et al., 2021; Meng et al., 2020; Shi et al., 2021; Shu et al., 2020). Our study applied not only hUC-MSCs but also cells overexpressing ACE2, and the results are consistent with previous studies. To test the effects of ACE2-MSCs on COVID-19 treatment, we cocultured ACE2-MSCs with LPS-treated AT-II/-S or Beas-2B/-S cells. The inflammatory factors *TNF-α*, *IL-1β*, *IL-6*, *IL-8*, and *IL-18* were all repressed by ACE2-MSCs treatment in AT-II and AT-II-S cells compared with treatment with MSCs alone, and most of the examined pyroptosis factors were also decreased by ACE2-MSCs application (Figure 3D). Interestingly, in spike protein-transfected AT-II cells, ACE2-MSCs strongly inhibited all these genes compared with GFP-MSCs. Similar results were obtained in LPS-treated Beas-2B/-S cells, but the repression range of ACE2-MSCs to each gene was quite narrow compared with that in LPS-treated AT-II/-S cells (Figure 3E). Thus, we speculate that ACE2-MSCs might have different regulatory effects on two different types of cells. In SARS-CoV-2 pseudovirus-infected hACE2 transgenic mice, ACE2-MSCs and GFP-MSCs also repressed key members of a cytokine storm (such as *TNF-α*, *INF-γ*, *IL-10*, *IL-15*, *IL-17*, and *IL-1β*) and pyroptosis factors *GSDMD*, *Casp1*, *Casp4*, *NLRP3*, and *NLRC4* compared with the PBS treatment group (Figure 5F). Clinical-grade soluble human ACE2 was reported to have the capacity to inhibit COVID-19 infections and repress the transcript levels of proinflammatory factors (TNF-α, IL-1β, and IL-6) (Monteil et al., 2020). ACE2-overexpressing A549 cell-derived microparticles showed potential therapeutic effects against SARS-COV-2 infection (Wang et al., 2022). In the bleomycin-induced lung injury, ACE2 alone affected the apoptosis factors but weaker than stem + ACE2 (Zhang et al., 2015). In the sepsis-induced acute lung injury, recombinant human ACE2 protein attenuated lung injury (Imai et al., 2005). Consistent with the previous reports, we found that using rh-ACE2 alone could attenuate the lung injury and repress some inflammatory factors *TNF-α*, *INF-γ*, *IL-10*, *IL-17*, and *IL-1β* (Figure 5F). However, compared with the therapeutic effects of ACE2-MSCs, rh-ACE2 showed weaker ones, probably because ofthe shorter effect lasting time with rapid metabolism. ACE2-MSCs only showed better therapeutic effects on *IL-15*, *IL-17*, *IL-1β*, *GSDMD*, and *Casp1* than GFP-MSCs. Therefore, based on the *in vivo* and *in vitro* results, we speculate that ACE2-MSCs are much more effective in treating COVID-19 patients than GFP-MSCs or rh-ACE2 alone. However, the molecular mechanisms underlying this process are poorly understood and will be the focus of upcoming studies.

In conclusion, in COVID-19-infected cells or animals, MSCs modified with the ACE2 gene or even other vital gene(s) will be a promising approach for the treatment of COVID-19 patients. In addition, targeting core members of the cytokine storm or pyroptosis in therapy provides some new strategies for developing new treatments for COVID-19 patients.

### Limitations of the study

This study has potential limitations. Our results demonstrated that the therapeutic effects of ACE2-MSCs are based on SARS-CoV-2 pseudovirus and spike protein of SARS-CoV-2 induced lung injury *in vivo* and *in vitro*. Even though the spike protein of SARS-CoV-2 has been used to induce lung injury (Kuba et al., 2005) and helped develop an RBD-Fc-based COVID-19 vaccine (Liu et al., 2020), pseudoviruses expressing the SARS-CoV-2 spike protein could infect target cells without replication, and therefore have been used widely as an alternative strategy for studying COVID-19 treatment (Liu et al., 2020; Xia et al., 2020a, 2020b) — the results partially mimic the real COVID-19. Therefore, the therapeutic effects of ACE2-MSCs on the current COVID-19 models may not be solid enough for clinical research. Further research with real SARS-CoV-2 virus infection is required to evaluate the therapeutic effects of ACE2-MSCs on COVID-19 and to better understand the mechanism through which ACE2-MSCs modulate cellular and signaling networks in response to microenvironmental cues in COVID-19. On the clinical side, regarding optimal therapeutic doses and optimal route(s) of administration of ACE2-MSCs should be the primary concern. Recently, WHO warned that with the rapid transmission and spread of Omicron in global area, the high infection rate might precipitate the emergence of new variant strain(s); people may never be able to vanquish the SARS-CoV-2 virus and it will eventually become part of the ecosystem. Thus, the prevention and treatment of COVID-19 are particularly important and urgent. Our findings provide some new strategies for COVID-19 treatment and have some clinical significance. We could hardly wait to share the current results and help people defeat the SARS-CoV-2 virus.

## STAR★Methods

### Key resources table

**Table:** 

REAGENT or RESOURCE	SOURCE	IDENTIFIER
**Bacterial and virus strains**		

SARS-CoV-2 spike pseudovirus	OBiO Technology Co. Ltd., Shanghai, China	H7657
HBLV-ZsGreen-PURO	Hanbio Biotechnology Co. Ltd., Shanghai, China	lv44061917
HBLV-h-ACE2-3xflag-ZsGreen-PURO	Hanbio Biotechnology Co. Ltd., Shanghai, China	lv44061918

**Experimental models: Cell lines**		

Human: AT-II cells	Bluefbio Biology Technology Development Co., Ltd. Shanghai, China	BFN6080397
Human: Beas-2B cells	Bluefbio Biology Technology Development Co., Ltd. Shanghai, China	BFN608009328; RRID: CVCL_UR57
Human: HUC-MSCs cells	Genesis Stem-cell Co. Ltd.	N/A

**Experimental models: Organisms/strains**		

hACE2 transgenic mice	Beijing Viewsolid Biotech Co. Ltd.	Tg ICR-Ace2(+)/V

**Oligonucleotides**		

Primers for PCR, see Table 1	This paper	N/A

**Recombinant DNA**		

S protein plasmid	National center for Protein Science (Shanghai, China)	NFPS-P10032 pCDNA3.4-S

**Chemicals, Peptides, and Recombinant Proteins**		

SARS-CoV-2 (2019-nCoV) Spike RBD-Fc Recombinant Protein (HPLC-verified)	Sino Biological (Beijing, China)	40592-V02H
Recombinant Human ACE-2 Protein (rh-ACE2), CF	R&D Systems	933-ZN-100
Lipopolysaccharide (LPS)	Sigma-Aldrich, Missouri, USA	MFCD00164404
0.25% Trypsin-EDTA	Gibco, New York, USA	2277047
Penicillin/Streptomycin/Savelt anti-mycoplasma reagent (PSS)	Hanbio (Shanghai, China)	HB-PSS-100
Lipofectamine™ 2000 CD Transfection Reagent	Thermo fisher scientific, MA, USA	12566014
G418 (Geneticin)	Thermo fisher scientific, MA, USA	10131035
Puromycin	Thermo fisher scientific, MA, USA	A1113802
Fetal Bovine Serum (FBS)	Gibco, New York, USA	1715753
RNA-Quick Purification Kit	Jiangsu Real-gen Biotechnology Co., Ltd., Suzhou, China	RN001
HiScript II Q Select RT SuperMix for qPCR	Vazyme, Nanjing, China	R233-01
2 × Taq Master Mix (Dye Plus)	Vazyme, Nanjing, China	P112-01
TaKaRa TB Green™ Premix Ex Taq™ II (Tli RNaseH Plus)	Takara Biomedical Technology (Beijing) Co., Ltd., Beijing, China	RR420
BCA protein assay kit	Beyotime, Shanghai, China	P0012
Mouse direct PCR kit	Bimake, Houston, USA	B40015
Angiotensin II Converting Enzyme (ACE2) Activity Assay Kit (Fluorometric)	Biovision, San Francisco, USA	K897-100
Cell counting kit-8 (CCK8)	Beyotime, Shanghai, China	C0038

**Other**		

DMEM Without Sodium Pyruvate	Meilunbio, Dalian, China	MA0560
RMPI 1640	Corning, New York, China	10040023

**Software and algorithms**		

GraphPad Prism	GraphPad Software, San Diego, USA	GraphPad Prism 6; RRID: SCR_002798
Opti-MEM	Gibco, New York, USA	31985070


The data generated by our analysis can be downloaded from the sources listed in the Table S1.

### Resource availability

#### Lead contact

Further information and requests for resources and reagents should be directed to and will be fulfilled by the lead contact, Gang Chen (chengang6626@ntu.edu.cn).

#### Materials availability

This study did not generate new unique reagents.

### Experimental model and subject details

#### Animals

hACE2 transgenic mice were purchased from Beijing Viewsolid Biotech Co. Ltd. (constructed by Institute of Laboratory Animal Science, Peking Union Medical School) (Bao et al., 2020; Yang et al., 2007). hACE2 transgenic adult mice (8–10 weeks, both sexes, 30–35 g) were used for acute lung injury (ALI) model construction and SARS-CoV-2 pseudovirus infection. All mice were raised with food and water available *ad libitum* under a 12-h light/dark cycle, and each cage housed two to five mice. All applicable institutional and/or national guidelines for the care and use of animals were followed. Animal studies were *performed according to guidelines established by the Institutional Animal Care and Use Committee of Nantong University* (permission number: S20200013-025) *and were conducted following the ARRIVE guidelines*.

#### Cell culture

AT-II cells (BFN6080397) and Beas-2B cells (BFN608009328, RRID: CVCL_UR57) were purchased from BFB BluefBio. hUC-MSCs were donated by Genesis Stem-cell Co., Ltd. Cells were grown in complete medium containing 90% basic medium, 10% FBS, and 1% PSS. The basic media for AT-II and Beas-2B cells were pyruvic acid-free DMEM and RPMI 1640, respectively. hUC-MSCs culture medium was provided by Genesis Stem-cell Co., Ltd. All the cells were cultured in an incubator at 37°C, 5% CO_2_ and 95% air.

### Method details

#### Spike protein-transfected AT-II cells and Beas-2B

NFPS-P10032 pCDNA3.4-S plasmid, which contains the spike glycoprotein of SARS-CoV-2, was donated by the National Center for Protein Science Shanghai. A total of 1.5 μg plasmid was mixed with 80 μL pure 1,640 medium and incubated for 5 min. Then, 2 μL Lipofectamine™ 2000 (11668030, Thermo Fisher) was added and incubated for 20 min. The mixture was dripped into AT-II or Beas-2B cells for transfection. G418 (600 μg/mL) was applied to select the positive cells, and 300 μg/mL G418 was used to maintain the stable transfection lines.

#### Construction of ACE2-overexpressing hUC-MSCs

HBLV-h-ACE2-3xflag-ZsGreen-PURO lentivirus (lv44061918) containing the full-length coding sequences of ACE2 and HBLV-ZsGreen-PURO lentivirus (lv44061917) were purchased from HANBIO. BLV-h-ACE2-3xflag-ZsGreen-PURO or HBLV-ZsGreen-PURO lentivirus (50 MOI) with 6 μg/mL polybrene was dripped into hUC-MSCs for transfection. Puromycin (4.5 μg/mL) was applied to select the positive cells, and puromycin (2 μg/mL) was used to maintain the stable transfection lines.

#### Cell proliferation activity

AT-II, AT-II-S, Beas-2B, Beas-2B-S cells, Spike RBD-Fc recombinant protein-infected AT-II, AT-II-S, Beas-2B, or Beas-2B-S cells were plated in 96-well plates at 2000 cells per well. Four hours after planting, 10 ng/mL, 100 ng/mL or 5 μg/mL SARS-CoV-2 (2019-nCoV) spike RBD-Fc recombinant protein (HPLC-verified) was added to AT-II cells and Beas-2B cells and then cultured for certain hours. Four wells for each time point (4, 12, 36, 60, and 84 h) from each cell line were sampled for CCK-8 measurement. Briefly, the culture medium was removed, 90 μL 1,640 basic medium with 10 μL CCK-8 was added, and the cells were cultured in the dark for 1.5 h. OD450 was read by enzymatic reader.

#### Coculture system

AT-II and AT-II-S coculture systems were plated at 2∗10^5^ cells/well; Beas-2B and Beas-2B-S cells were plated at 3∗10^5^ cells/well, all in 6-well plates. Twelve hours later, all the cells were treated with LPS (2 μg/mL). ACE2-MSCs or GFP-MSCs (2∗10^5^ cells/well) were plated in a 0.45 μm Transwell membrane in a 6-well plate. After 24 h, AT-II/-S or Beas-2B/-S culture medium with LPS was replaced by Opti-MEM, and ACE2-MSCs or GFP-MSCs cultured in transwell membrane were put on the uperside. Cells were cocultured for 24 h, and then the cells were collected for the next experiment.

#### Cell apoptosis analysis

Flow cytometry analysis with Annexin V Alexa Fluor 647/7-AAD. A total of 2∗10^5^ AT-II/-S cells/well or 3∗10^5^ Beas-2B/-S cells/well in 6-well plates were cultured for 36 h, and then the cells were collected. After centrifugation, the cells were resuspended in binding buffer and adjusted to 1–5×10^6^ cells/mL. Then, 100 μL of cell suspension was transferred to a 5 mL flow tube, and 5 μL of Annexin V/Alexa Fluor 647 was added. After mixing and incubation for 5 min at room temperature in the dark, 10 μL 20 μg/mL 7-AAD and 400 μL PBS were added to immediately perform the flow detection. Three individual wells were sampled from each group, and each well was measured at least 3 times.

#### ACE2 enzyme activity

ACE2 enzyme activity was detected by an angiotensin II converting enzyme (ACE2) activity assay kit (Fluorometric) (Biovision, Catalog #K897-100). ACE2 lysis buffer was used to spill ACE2-MSCs or GFP-MSCs, and protein was acquired after being placed on ice, vortexed and centrifuged. A BCA kit (Beyotime, P0012) was used to measure the protein concentration. Five microliters of lysis product were added to each well in a 96-well plate, and the volumes of sulfur, hydrogen bromide, nitrocellulose, and polycarbonate were adjusted to 50 μL/well with ACE2 detection buffer, blended evenly and hatched for 15 min. Added 48 μL ACE2 detection buffer and 2 μL samples to every sample, Background Control, Positive Control and Negative Control well, measured fluorescence in kinetic mode (Ex/Em = 320/420 nm) from 30 min to 2 h. Two time points were selected randomly within the linear range of the graph (T1 and T2), and the corresponding fluorescence value was obtained. Then, ▵RFU/▵T5 was calculated.

#### LPS-induced ALI mice model

h-ACE2 transgenic mice were anesthetized with isoflurane (3 mL/min). LPS (*Escherichia coli*, O111: B4, Sigma) (10 mg/kg) (Yang et al., 2020) in 50 μL normal saline was administered by intratracheal injection, while the control group was given 50 μL sterile normal saline.

#### SARS-CoV-2 pseudovirus-infected mice

We administered SARS-CoV-2 pseudovirus (OBiO Scientific Service, H7657) 5∗10^5^ TU to every mouse by intratracheal injection to simulate a COVID-19-infected model. Pseudoviruses are chimeric virus particles that use lentiviruses modified from HIV as basic vectors, but the VSVG membrane protein of lentiviruses is replaced by the SARS-CoV-2 spike protein. The envelope is the SARS-CoV-2 spike protein, which carries a luciferase reporter gene. It can be used to infect cells expressing ACE2.

#### Intravenous tail injection

ACE2-MSCs or GFP-MSCs were collected, diluted to 5∗10^5^ cells/150 μL PBS and stored on ice. Recombinant human ACE-2 (rh-ACE2) protein (CF, R&D Systems, 933-ZN-100) was administrated at a dosage of 0.1 mg/kg (Imai et al., 2005). The mouse tail was fixed with a visual mouse tail intravenous injection device, and 150 μL suspensions was injected intravenously (He et al., 2015a). For each group, 4 to 6 mice were sacrificed. Lung tissues were collected for lung histological analysis and gene expression analysis after 36 hours of MSCs treatment (Gupta et al., 2007).

#### Histochemical analysis

Mice were dissected after deep anesthesia (isoflurane, 3 mL/min) and perfused with 0.9% NaCl solution through the left ventricle while the right atrial appendage was cut. After the effluent was clarified, the mice were perfused with 4% paraformaldehyde until the body was completely stiff. Lung specimens were harvested and fixed in 4% paraformaldehyde for 48 h. Then, dehydrated and embed lung specimens. 7 μm paraffin section was sliced up. After gradient dewaxing and staining with a hematoxylin and eosin stain kit (Beyotime, C0105S), histopathologic examination was performed.

#### Lung histological analysis

Stained sections were imaged by a Leica microscope. Choose 3 to 6 visions under 400x randomly to score obey the following standards set as pervious report (He et al., 2015a).

#### PCR and qRT–PCR

Regular PCR experiments were used to identify the transfection of SARS-CoV-2 spike protein in AT-II and Beas-2B cells and the genotyping of hACE2 mice. 129 bp PCR product and DNA marker (Vazyme, MD101-01-AA) were used for agarose (VETEC, V900510-100G) gel electrophoresis to authenticate Spike protein were transfected successfully. To genotype hACE2 transgenic mice, tail tissue of 3-week-old mice was sampled. Genotyping was performed using a mouse tail rapid gene identification kit (Bimake, B40015).

qRT–PCR was used to examine the gene expression levels of the main factors of the inflammatory response, pyroptosis, and cytokine storm in AT-II/-S and Beas-2B/-S cells and mouse lung tissue. AT-II and AT-II-S genes were plated at 2∗10^5^ cells/well; Beas-2B and Beas-2B-S cells were plated at 3∗10^5^ cells/well, all in 6-well plates. Cells were cultured for 12 h and then stimulated with LPS (2 μg/mL) for 24 h. Control group with no treatment. MSCs were cocultured with AT-II/-S or Beas-2B/-S cells for 24 h. Three wells of each group were collected for RNA extraction with RNA quick extract kit (ES Science, RN001). One ug RNA were used for reverse transcription with HiScript® III RT SuperMix (Vazyme, R323-01). qRT-PCR was performed with 100 ng cDNA, 10 uM primers, and 12.5 μL TB Green Premix Ex Taq (Tli RNaseH Plus) (2X) (Takara, RR420) (Chen et al., 2017). All the primers that were used in this study shown in the Table 1.

#### Immunohistochemical

After the mouse ALI model was established and MSCs-GFP cells were tail injected for 12, 24, 36, 60 and 84 h, the mice were perfused under deep anesthesia. Lung specimens were removed, postfixed in 4% paraformaldehyde for 9 h and dehydrated in 50% sucrose for more than 2 days. Lungs embedded with optimum cutting temperature compound (OCT, USA) were sectioned into 12 μm thick sections in a cryostat, adsorbed on glass slides and allowed to dry naturally. Then, the cells were sealed with anti-fluorescence quenching sealing solution (Beyotime, P0133) and imaged by laser scanning confocal microscopy (TCS SP8, Leica) (Chen et al., 2017).

#### Scanning electron microscope (SEM)

For SEM observation, in 24-well plate with glass slide in each well, AT-II and AT-II-S were planted 5∗10^4^ cells/well, 3 wells; Beas-2B and Beas-2B-S were planted 3∗10^4^ cells/well, 3 wells. The cells were cultured for 36 h, washed with PBS for 10 min 3 times and fixed with glutaraldehyde for 2 h and osmic acid for 2 h. Then, the cells were dehydrated using graded ethanol followed by isoamyl acetate treatment and coated with gold (thickness ∼50 nm). Finally, the coated samples were observed by SEM under a vacuum degree of 1.33 × 10-4 Pa at an acceleration voltage of 20 kV by S-3400N II (Hitachi, Japan).

### Quantification and statistical analysis

#### Statistical analysis

All data are expressed as the mean ± SD, as indicated in the figure legends. Student’s *t* test (two groups), one-way ANOVA or two-way ANOVA was used to compare the differences between groups, followed by Bonferroni’s test. The p values represented in each figure are shown in the figure captions. Statistical report was shown in Table S1 which related to all the figures. These data were analyzed by Graphpad Prism (RRID: SCR_002798).

## Data Availability

Data reported in this paper will be shared by the lead contact upon request. This paper does not report original code. Any additional information required to reanalyze the data reported in this paper is available from the lead contact upon request.

## Supplemental information

Document S1. Figures S1–S4

Table S1. Statistical report, related to all the figures and STAR methods
