# Supplementary material for: ACE2 overexpressing mesenchymal stem cells alleviates COVID-19 lung injury by inhibiting pyroptosis
Source: iScience. 2022 Mar 10;25(4):104046. doi: 10.1016/j.isci.2022.104046 (PMC8907105; doi:10.1016/j.isci.2022.104046)
Supplement: Document S1. Figures S1–S4 [file mmc1.pdf]

iScience, Volume *25*

Supplemental information

ACE2 overexpressing mesenchymal stem cells alleviates COVID-19 lung injury by inhibiting pyroptosis

Jinhuan Wei, Rui Shang, Jiaqi Wang, Shengze Zhu, JianQiang Yin, Ying Chen, Yayu Zhao, and Gang Chen

Supplementary Figure 1

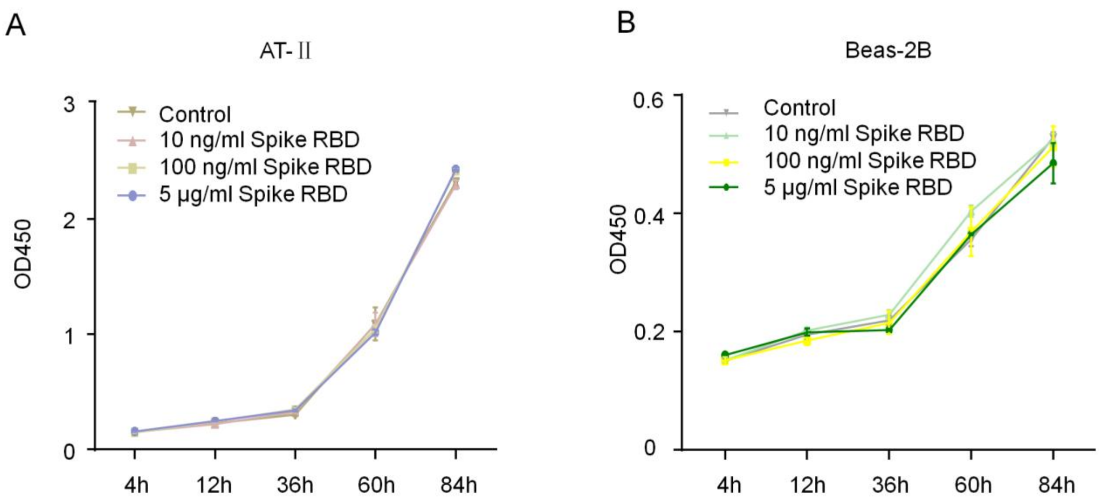

Figure S1: Cell growth of spike RBD-Fc recombinant protein-infected cells was measured by CCK-8, related to STAR Methods: Cell proliferation activity.

Measurement of cell proliferation of AT-II (A) or Beas-2B (B) treated with Spike RBD-Fc Recombinant Protein by CCK-8 4 hours (4 h), 12 hours (12 h), 36 hours (36 h), 60 hours (60 h), and 84 hours (84 h) after cell planking compared with the control group. SARS-CoV-2 (2019-nCoV) spike RBD-Fc recombinant protein (10 ng/ml, 100 ng/ml and 5 μg/ml) was applied to AT-II or Beas-2B cells at the 4-hour time point. There was no significant change in cell growth among the different dosages compared with the control group. Each time point we measured 4 replicate cell wells. All data are expressed as the mean ± SD, and Student’s t-test was used to compare the differences between groups, followed by Bonferroni’s test.

Supplementary Figure 2

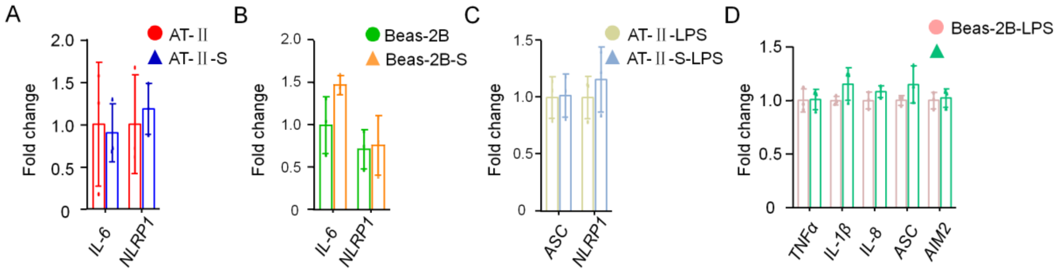

Figure S2: mRNA expression of those genes that were not affected by spike protein transfection or LPS treatment, related to Figure 2.

(A, B) *IL-6* and *NLRP1* were not affected by spike protein transfection in either AT-II cells or Beas-2B cells. (C) *ASC* and *NLRP1* in AT-II and AT-II-S cells were not changed by LPS treatment. (D) There was no significant change in the transcript levels of *TNFα*, *IL-1β*, *IL-8*, *ASC* and *AIM2* in Beas-2B and Beas-2B-S cells treated with LPS. The relative expression of target genes was normalized to *18S* rRNA. All data are expressed as the mean ± SD, N=3 per group, and Student's t-test was used to compare the differences between groups, followed by Bonferroni's test.

Supplementary Figure 3

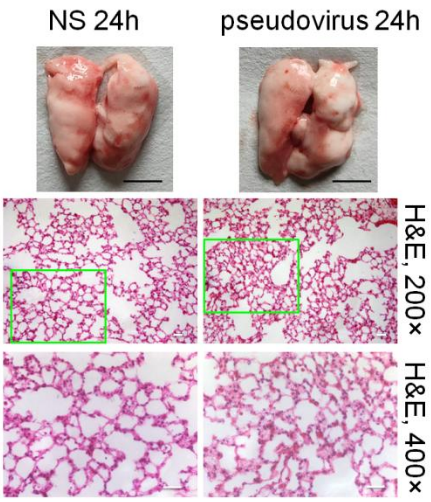

Figure S3: Histological evaluation of lungs from SARS-CoV-2 pseudovirus-infected mice, related to Figure 4.

Fifty microliters of normal saline (NS) or pseudovirus ($5*10^5$ TU/mouse) was intratracheally injected. Twenty-four hours later, lung tissues were collected for histological evaluation. Regardless of gross histological tissue or HE-stained lung tissue, no detectable alteration was shown. Gross pathology and histopathology of lung tissues from NS 24 h and SARS-CoV-2 pseudovirus 24 h are shown. Scale bars: white, 0.5 cm; black, 50 μm.

Supplementary Figure 4

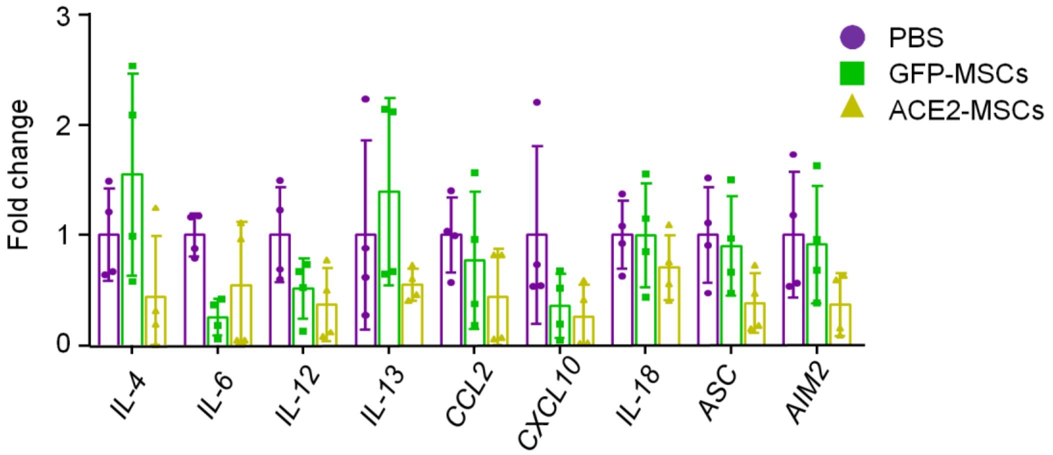

Figure S4: mRNA expression of those genes that were not affected by MSCs administration, related to Figure 5.

Neither the transcript levels of main factors of cytokine storm: *IL-4*, *IL-6*, *IL-12*, *IL-13*, *CCL2*, *CXCL10* and *IL-18*, nor that of the key pyroptosis genes *ASC* and *AIM2* were changed by the administration of GFP-MSCs or ACE2-MSCs. The relative expression of target genes was normalized to *18S* rRNA. All data are expressed as the mean ± SD, N=4 per group, and one-way ANOVA was used to compare the differences between groups.
